# Allelopathic Potential of *Marsdenia tenacissima* (Roxb.) Moon against Four Test Plants and the Biological Activity of Its Allelopathic Novel Compound, 8-Dehydroxy-11*β*-*O*-Acetyl-12*β*-*O*-Tigloyl-17*β*-Marsdenin

**DOI:** 10.3390/plants12081663

**Published:** 2023-04-15

**Authors:** Seinn Moh Moh, Naoaki Kurisawa, Kiyotake Suenaga, Hisashi Kato-Noguchi

**Affiliations:** 1 Department of Applied Biological Science, Faculty of Agriculture, Kagawa University, Miki, Kagawa 761-0795, Japan; 2 The United Graduate School of Agricultural Sciences, Ehime University, Matsuyama 790-8566, Japan; 3 Department of Chemistry, Faculty of Science and Technology, Keio University, Kohoku, Yokohama 223-8522, Japan

**Keywords:** *Marsdenia tenacissima*, allelopathic substances, growth inhibition, novel compound (steroidal glycoside 3;8-dehydroxy-11*β*-*O*-acetyl-12*β*-*O*-tigloyl-17*β*-marsdenin)

## Abstract

Plant parts and extracts that are rich in bioactive substances with allelopathic potential can be explored as a possible alternative to herbicides for natural weed control in sustainable agriculture. In the present study, we investigated the allelopathic potential of *Marsdenia tenacissima* leaves and its active substances. Aqueous methanol extracts of *M. tenacissima* showed significant inhibitory activities against the growth of lettuce (*Lactuca sativa* L.), alfalfa (*Medicago sativa* L.), timothy (*Phleum pratense* L.), and barnyard grass (*Echinochloa crusgalli* (L.) Beauv.). The extracts were purified through various chromatography steps, and one active substance was isolated and determined by spectral data to be a novel compound, assigned as steroidal glycoside 3 (8-dehydroxy-11*β*-*O*-acetyl-12*β*-*O*-tigloyl-17*β*-marsdenin). Steroidal glycoside 3 significantly inhibited the seedling growth of cress at a concentration of 0.03 mM. The concentrations needed for 50% growth inhibition of the cress shoots and roots were 0.25 and 0.03 mM, respectively. These results suggest that steroidal glycoside 3 may be responsible for the allelopathy of *M. tenacissima* leaves.

## 1. Introduction

Weeds inflict serious restrictions on agricultural production [1]. They reduce agricultural productivity and quality because both weeds and crops compete for natural resources through allelopathy [2] and parasitism [3], and they also harbor pests and plant pathogens [4]. Weeds have been controlled either mechanically or by using herbicides [5]. Among weed control methods, herbicide application is the most effective. Therefore, agricultural weed control relies heavily on herbicides [6]. However, the overuse of synthetic agrochemicals for weed control has increased environmental pollution, unsafe agricultural products, and human health concerns [7], and resulted in the evolution of herbicide-resistant weeds [8]. Consequently, the negative effects of commercial herbicide use on the environment make it desirable to diversify weed management options [9,10,11]. Many investigations have attempted to exploit the allelopathy of plants for weed control purposes [12,13] because allelopathic plants are used as cover crops, green manure, and mulch, and they can help to reduce noxious weeds and improve crop production and soil quality [14]. In addition, allelochemicals, or secondary metabolites with phytotoxic effects, have been discovered in a range of plants, so these allelochemicals have been investigated as potential candidates for herbicides [15,16,17]. Therefore, allelopathic plants and their allelochemicals could be useful for weed management options in a variety of agricultural settings, potentially reducing dependency on commercial herbicides [13,18]. Nowadays, allelopathic plants and allelochemicals that have the biological ability to suppress weeds are receiving the most attention. For example, Kato-Noguchi et al. (2014) [19] discovered that two novel compounds, nimbolide B and nimbic acid B, from the leaves of *Azadirachta indica* have strong allelopathic effects on the growth of cress and barnyard grass; leaf extracts of *Dregea volubilis* have phytotoxic potential against timothy, barnyard grass, lettuce, and alfalfa, and the phytotoxic activity of its two compounds, 3-hydroxy-α-ionone and 5-hydroxy-3,4-dimethyl-5-pentylfuran-2(5*H*)-one, were successful against two test plants [20]; and the allelopathic effects of two phenolic chemicals, vanillic acid and ferulic acid, which were isolated from the leaves of *Senna garrettiana*, on the growth of cress were described by Krumsri et al. in 2022 [21]. Based on these findings, many plant species could contain bioactive substances with potential allelopathic effects.

**Figure 1:**
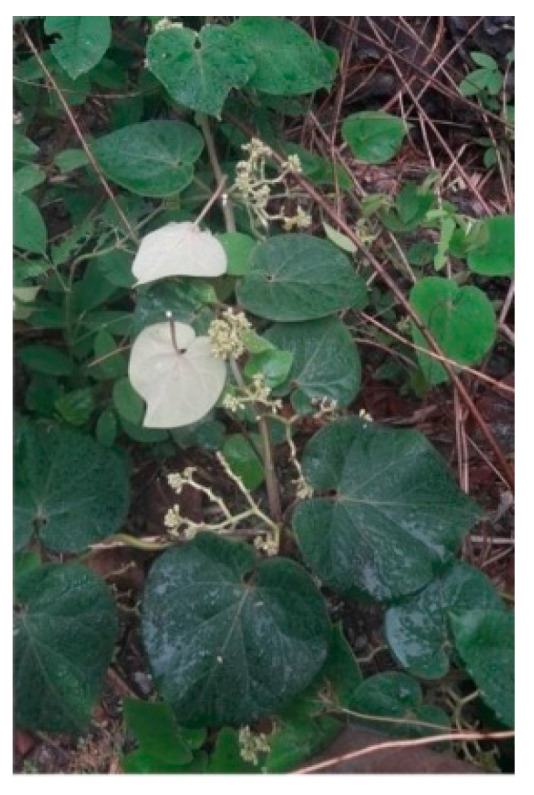
*Marsdenia tenacissima*.

*Marsdenia tenacissima* (Roxb.) Moon (family: *Asclepiadaceae*) is a perennial climber extensively distributed in the tropical and subtropical parts of Asia. This species can be found below an altitude of 2000 m in dry and damp deciduous forests with an annual rainfall of 1000–1500 mm [22]. Its leaves are opposite and heart-shaped (Tiwari et al., 2018) [23]; the inflorescences are greenish yellow, numerous, and large, arranged in many branches; and the roots are cylindrical and yellow to buff [24] (Figure 1). The roots of *M. tenacissima* have been widely used as a herbal medicine by the Dai people who reside in Laos, Myanmar, and Yunnan province in China [25]. Different parts of the *M. tenacissima* plant have been used in traditional medicine to treat various ailments such as pneumonia, cancer, fever, cough, vomiting, tumors, diabetes mellitus, heart disease, postpartum milk impassability in women, and gonorrhea [24,26]. A phytochemical investigation of this plant has reported that it contains 196 phytochemicals, including 155 steroids, triterpenes, phenolic compounds, and organic acids (Wang et al., 2018) [22]. In addition, the leaves contain benzoic acid, gallic acid, pyrogallol, salicylic acid, trans-cinnamic acid, and vanillic acid [27]. Pharmacological studies have revealed that this plant possesses anti-cancer [28,29], anti-HIV [30], anti-tumor [31], anti-inflammatory, anti-diarrheal, and immunomodulatory properties [32]. Many researchers have extensively studied the phytochemical constituents and pharmacological activities of different parts of this plant. However, there is little information on its allelopathic activity and substances. In our previous research, we discovered that *M. tenacissima* extracts significantly restricted the growth of Italian ryegrass and cress, and we also identified two phytotoxic compounds in its extracts. Additionally, the other active peak from the same fraction of *M. tenacissima* has been found to have strong phototoxic activity, indicating that we can isolate that other bioactive substance from its extracts [33]. Therefore, the current study was undertaken to determine the allelopathic potential of *M. tenacissima* against the growth of four test plants, to identify the allelopathic substances from its extract, and to assess their biological activities.

## 2. Results

### 2.1. Allelopathic Activity of the Marsdenia tenacissima

**Figure 2:**
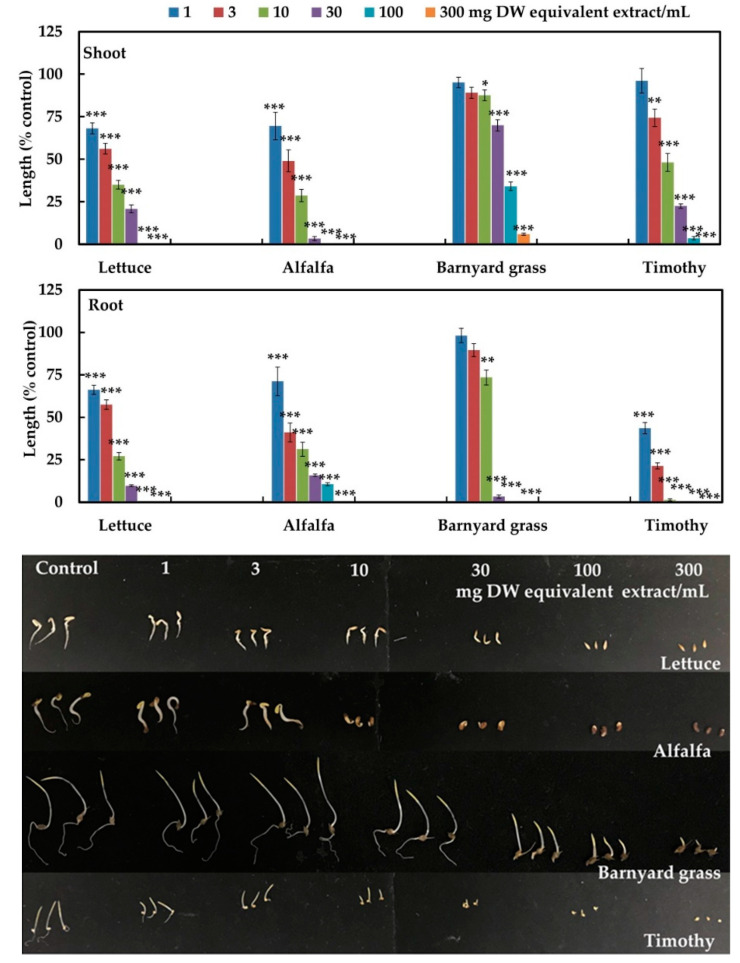
Effects of aqueous methanol extracts of *Marsdenia tenacissima* leaves on the root and shoot growth of lettuce, alfalfa, barnyard grass, and timothy with the concentrations corresponding to the extracts acquired from 1, 3, 10, 30, 100, and 300 mg DW equivalent extract/mL. The bars on each experiment show mean ± SE from two independent experiments with three replications and 10 plants for each treatment (*n* = 60). Asterisks indicate significant difference between control and treatment: * *p* < 0.05, ** *p* < 0.01, *** *p* < 0.001.

The leaf extracts of *M. tenacissima* suppressed the seedling growth of lettuce, alfalfa, barnyard grass, and timothy at concentrations greater than 3 mg DW equivalent extract/mL, (*p* < 0.05) (Figure 2). The leaf extracts obtained from 10 mg of *M. tenacissima* inhibited the shoot growth of lettuce, alfalfa, barnyard grass, and timothy to 35, 28.72, 87.56, and 48.08% of the control, respectively, whereas the root growth was inhibited to 27.07, 31.25, 73.47, and 1.36% of the control, respectively, (*p* < 0.001). Moreover, the extract concentration of 300 mg DW equivalent extract/mL completely inhibited the shoot and root growth of the lettuce, alfalfa, and timothy, and the root growth of barnyard grass, but not its shoots, compared with the control.

The *I_50_* values of the *M. tenacissima* extracts for the shoot and root growth of lettuce, alfalfa, barnyard grass, and timothy varied from 0.7 to 54.2 mg DW equivalent extract/mL (Table 1). The *I_50_* values of the *M. tenacissima* extracts for the shoot growth of lettuce and alfalfa were not significantly different compared with its root growth, whereas the root growth of barnyard grass and timothy was significantly less than their shoot growth.

### 2.2. Isolation and Identification of the Active Substance

**Figure 3:**
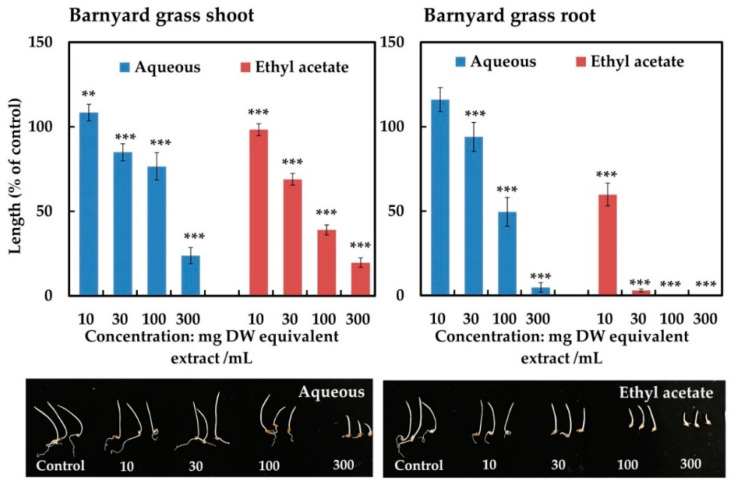
The inhibitory effect of the aqueous and ethyl acetate fractions obtained from *Marsdenia tenacissima* on the shoot and root growth of barnyard grass. The treatment concentrations were 10, 30, 100, and 300 mg DW equivalent extract/mL. The bars on each experiment express mean ± SE from three replicates, each with 10 seedlings (*n* = 30). Asterisks indicate significant difference between control and treatment: ** *p* < 0.01, *** *p* < 0.001.

The leaf extracts of *M. tenacissima* were separated through partitioning into ethyl acetate and aqueous fractions. Both fractions showed concentration-dependent inhibitory activity against the shoot and root growth of barnyard grass (Figure 3). To evaluate the biological activity of both fractions, barnyard grass (*Echinochloa crus-galli* (L.)) was selected as a representative monocot test plant because it was most sensitive to the plant extracts of the ethyl acetate fraction at high concentrations with regard to root development. At the concentration of 300 mg DW equivalent extract/mL, the ethyl acetate fraction inhibited the shoot growth of barnyard grass to 19.58% of the control and the root growth was completely inhibited, while the aqueous fraction inhibited the shoot and root growth to 23.79 and 4.72%, respectively (*p* < 0.001). Moreover, the ethyl acetate fraction at 10 mg DW equivalent extract/mL inhibited the root growth of barnyard grass more than the aqueous fraction. Therefore, the ethyl acetate fraction was selected for further purification and separated through a series of chromatography steps: silica gel, Sephadex LH-20, reverse-phase C_18_ cartridges, and HPLC. Finally, one active substance was isolated and characterized by reverse-phase HPLC and spectral data analysis.

**Figure 4:**
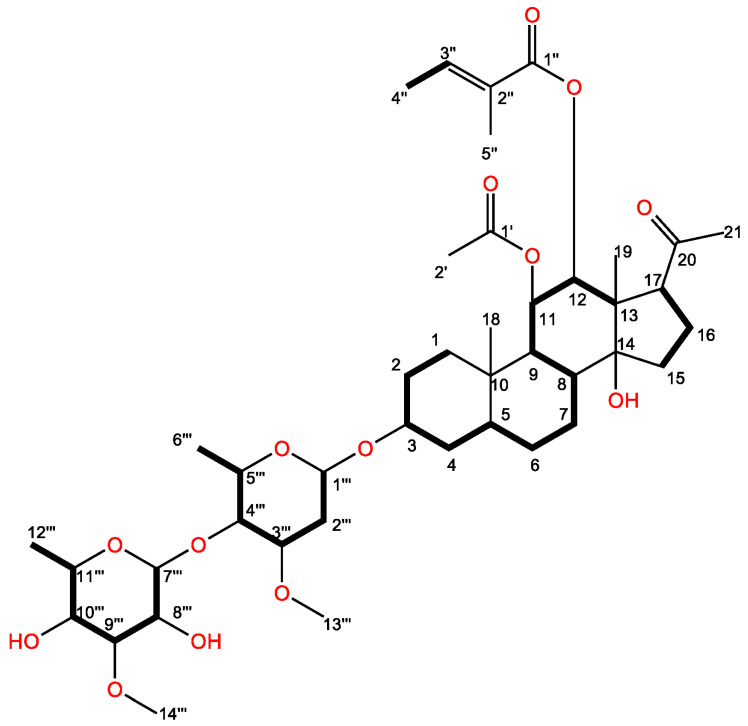
Molecular structure of the active compound.

The molecular structure of the characterized active compound was determined as C_42_H_66_O_14_ by using HR-ESIMS m/z 817.4355 [M + Na]^+^ (calcd for C_42_H_66_O_14_Na 817.4350) (Figure 4). The specific rotation of the compound showed [α] _D_^23^ = +31 (c 0.21, CH_3_OH); IR (neat) 3446, 2972, 2931, 2864, 1740, 1716, 1700, 1373, 1256 cm^−1^. The ^1^H and ^13^C-NMR data are summarized in Table 2.

### 2.3. Biological Activity of the Active Compound

**Figure 5:**
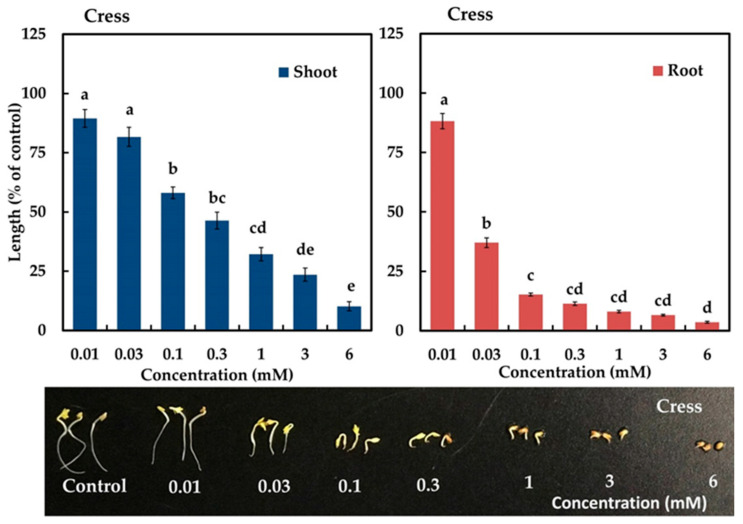
Inhibitory activity of the active compound on the shoot and root growth of cress. The bars on each experiment express mean ± SE from three replicates, each with 10 seedlings (*n* = 30). Different letters indicate significant differences among the treatments (Tukey’s HSD, *p* < 0.05).

The active compound significantly inhibited the seedling growth of cress (*Lepidium sativum* L.), and the degree of inhibitory activity increased with increasing concentration of the compound. The cress shoots and roots were significantly inhibited at concentrations greater than 0.1 and 0.03 mM, respectively (*p* < 0.05) (Figure 5). At a concentration of 6 mM, the shoot and root growth of cress was inhibited to 10.2 and 3.58% of the control, respectively. The *I_50_* values of the shoot and root growth of cress were 0.25 and 0.03 mM, respectively (*p* < 0.001).

## 3. Discussion

In our previous research, we found significant inhibitory effects of *M. tenacissima* extracts against the growth of cress and Italian ryegrass [33]. We evaluated the inhibitory effects of this extract against the growth of four other test plants (lettuce, alfalfa, barnyard grass, and timothy) to corroborate the previous findings. In the present research, the aqueous methanol extracts of the *M. tenacissima* leaves significantly inhibited the seedling growth of lettuce, alfalfa, barnyard grass, and timothy (Figure 2). The growth of the four test plants decreased with the increase in extract concentration, showing that the inhibitory effect depended on concentration. Such concentration-dependent inhibitory activity of several plant extracts has also been documented in other studies: those of Al-Harbi (2020) [34], Moh and Kato-Noguchi (2022) [35], Bari and Kato-Noguchi (2017) [36], Das and Kato-Noguchi (2018) [37], and Poonpaiboonpipat et al. (2021) [38]. Additionally, the *I_50_* values of the shoot and root growth of the four test plants varied, showing that the inhibition by the *M. tenacissima* extracts was species dependent (Table 1). A similar trend in concentration and species-dependent inhibitory activity for extracts of *Elaeocarpus floribundus*, *Anredera cordifolia*, *Garcinia xanthochymus*, and *Plumbago rosea* have also been reported [39,40,41,42]. Therefore, the *M. tenacissima* leaf extracts may contain allelopathic substances responsible for the growth-inhibitory activities against the four tested plants.

**Figure 6:**
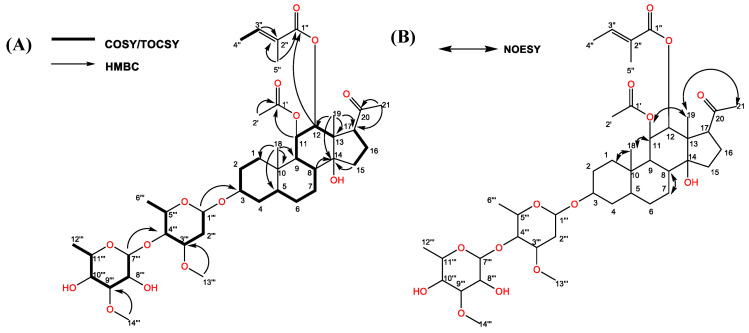
(**A**) COSY/TOCSY, HMBC and (**B**) NOESY correlations of the active compound.

The ethyl acetate fraction (partitioned from the aqueous methanol extracts of *M. tenacissima*) was separated on a silica gel column, and the allelopathic activity of each separated fraction was determined. The most active fraction was further purified by Sephadex LH-20, reverse-phase C_18_ cartridges, and HPLC, and the inhibitory activity was determined by a cress bioassay. One active compound was isolated and characterized through HR-ESIMS, ^1^H, and ^13^C-NMR. The aglycone moiety of the active compound had a polyoxypregnan-20-one skeleton according to a comparison of its ^1^H and ^13^C-NMR spectroscopic signals with those of marsdenosides A–H [43] and tenacissosides L and M [44], which was identified from the stem of *M. tenacissima*. Therefore, the structure of the active compound was elucidated based on the spectroscopic data of pregnane glycoside or C_21_ steroidal glycoside. The spectroscopic signals of the pregnane skeleton were assigned through analysis of ^1^H-^1^H COSY, TOCSY, HMBC, and NOESY spectra (Figure 6A,B). In the ^1^H and ^13^C-NMR spectra, we found that the two angular methyl groups at CH_3_-18 and CH_3_-19 (δ_H_ 0.94 (s), 1.04 (s); δ_C_ 12.6, 11.7) (Table 2); one tertiary methyl group at CH_3_-21 (δ_H_ 2.12 (s); δ_C_ 32); three oxygenated tertiary carbons at CH-3, CH-11, and CH-12 (δ_H_ 3.61, m 5.28, dd (10.1, 10.1), 4.86, d (10.1); δ_C_ 77.9, 72.5, 78.7); three quaternary carbons at C-10, C-13, and C14 (δ_C_ 38.7, 55.6, 85.2); two secondary methyl groups at CH_3_-6′″ (δ_H_ 1.35, d (5.9); δ_C_ 18.8) and CH_3_-12′″ (δ_H_ 1.23, d (6.3); δ_C_ 18.2); two methoxy groups at CH_3_-13′″ (δ_H_ 3.40 (s); δ_C_ 57.4) and CH_3_-14′″ (δ_H_ 3.59 (s); δ_C_ 62.5); and the ester carbonyl signals at C-1′ (δ_C_ 172.1) and C-1″ (δ_C_ 169.1) indicated that the active compound carried two acyl groups. An NMR study (^1^H and ^13^C-NMR, HMBC, TOCSY, and NOESY) and a consideration of the molecular structure of the active compound includes one carbonyl carbon of a ketone group, two acyl groups, five methyls, seven methylenes, six methines, three quaternary carbons (one oxygenated), two olefinic carbons, and dehydroxy alcohol, as well as two sugar units.

In the HMBC spectrum, the active compound showed a specific rotation system from H-1 to H-4 and H-9 to H-12, H-5 to H-8, and the correlations between H-15/H-16, which might be elucidated by the four-ring skeleton of a pregnane derivative. The HMBC correlations from H-18 to C-1, C-5, C-9, and C-10, and H-19 to C-12, C-13, C-14, and C-17 indicate the two angular methyl groups were connected to C-10 and C-13, respectively.

One additional methyl signal of CH_3_-2′ (δ_H_ 1.82 (s)) together with two carbon signals at δ_C_ 172.1 and 21.6 suggested one acetyl (Ac) group on the aglycone of the active compound. This group was attached at the C-11 position on the basis of HMBC correlations from CH-11 (δ_H_ 5.28, dd (10.1, 10.1)) to δ_C_ 172.1 (C-1′ of Ac) (Figure 6A,B). The tigloyl (Tig) group was identified through a series of proton signals at CH-3″ (δ_H_ 6.93, brq), CH-4″ (δ_H_ 1.85, brs), and CH-5″ (δ_H_ 1.85, s) with carbon resonance signals at δ_C_ 169.1, 129.1, 140.6, 14.6, and 12.1 in the ^13^C-NMR spectrum. The Tig group was attached at the CH-12 (δ_H_ 4.86, d (10.1)) position on the long-range of HMBC correlations from to δ_C_ 169.1(C-1″ of Tig). Moreover, the correlation from the protons CH-11 to CH-8 and CH-12 to CH-9 revealed that the Ac group at CH-11 and the Tig group at CH-12 were in β-orientation in the NOESY spectrum. Moreover, NOESY correlation between H-12 and C-17 (δ_H_ 2.89, dd (4.8, 9.6)) indicated that the C-17 side-chain was in β-orientation and the carbonyl carbon of the β-linked methyl ketone at C-17 appeared near δ_C_ 216.0. Similar findings of the β-linked methyl ketone at C-17 were discovered near δ_C_ 214.5 and δ_C_ 217 according to earlier studies [45,46].

In the ^1^H NMR spectrum, the two anomeric protons at CH-1′″ (δ_H_ 4.64, dd (1.6, 9.6)) and CH-7′″ (4.72, d (8.1)) indicated that the two sugar units were attached by β-linkage to the aglycone of the identified compound. The structures of the sugar units were fully determined by analysis of NMR data, including ^1^H-^1^H COSY/TOCSY, HMBC, and NOESY experiments (Figure 6A,B), and were further confirmed by comparison of the data with those in the literature [47]. Thus, the two sugar units were characterized as β-cymarose I (Cym I) and β-cymarose II (Cym II). We further assigned these proton signals based on analysis of its ^1^H-^1^H COSY correlations between CH-Cym II-7′″ (δ_H_ 4.72, d (8.1)) and CH- Cym II-8′″ (δ_H_ 3.31, m), CH-Cym II-8′″ (δ_H_ 3.31, m) and CH-Cym II-9′″ (δ_H_ 3.62, m), CH-Cym II-9′″ (δ_H_ 3.62, m) and CH-Cym II-10′″ (δ_H_ 3.17, m), CH-Cym II-10′″ (δ_H_ 3.17, m) and CH-Cym II-11′″ (δ_H_ 3.37, m), and CH-Cym II-11′″ (δ_H_ 3.37, m) and CH_3_-Cym II-12′″ (δ_H_ 1.23, d (6.3)). In addition, the HMBC spectrum revealed ^1^H-^13^C-NMR long-range correlations of CH-Cym I-1′″ to the C-3 of the aglycone (δ_C_ 77.9), and CH-Cym II-7′″ (δ_C_ 102.2) to CH-Cym I-4′″ (δ_C_ 84.0), which indicated that the sugar chain was attached at C-3 and the two sugars were linked through the 1→4 position. We also detected dehydroxy alcohol at the C-8 position from this active compound. Therefore, the structure of the active compound was determined to be a novel compound (steroidal glycoside 3) and defined as 3-*O*-[*β*-cymaropyranosyl (1→4)-*β*-cymaropyranosyl]-8-dehydroxy-11*β*-*O*-acetyl-12*β*-*O*-tigloyl-17*β*-marsdenin.

Many researchers have reported that C_21_ steroidal glycosides possess a wide range of pharmacological activities [44,48,49,50,51], as well as allelopathic activities [33]. Additionally, Kenji et al. (1998) [52] mentioned that the steroid glucoside, which was isolated from *Vernonia indica* S. Moore, exhibits seedling growth inhibitory activities on lettuce (*Lactuca sativa* L.). However, our report is the first on the allelopathic potential of steroidal glycoside 3 from *M. tenacissima*.

In this study, steroidal glycoside 3 significantly suppressed the shoot and root growth of the cress seedlings (Figure 5) and the inhibitory activity varied with compound concentration. Based on the *I_50_* values, steroidal glycoside 3 inhibited the roots more than the shoots. Previous reports also confirmed that root growth is more sensitive to allelochemicals than shoot growth [20,33]. This inhibitory activity of steroidal glycoside 3 might be due to the different molecular structures [53] and acyl moieties in the C-11 and C-12 positions [47]. Panda et al. (2006) [54] reported that pregnane glycosides with acyl moieties at C-11 or C-12, such as acetyl, benzoyl, and cinnamoyl, are more active. Steroidal glycoside 3 (in this study) and steroidal glycoside 1 (in the previous study) [33] have the same acyl moieties at the C-11 or C-12 position and sugar group, but they differ in the presence of dehydroxy alcohol in steroidal glycoside 3 and a hydroxy group in steroidal glycoside 1 at the C-8 position. Furthermore, the *I_50_* values of shoot growth of xxxsteroidal glycoside 3 exhibited higher allelopathic potential than steroidal glycoside 1. These two steroidal glycosides, however, showed greater growth inhibitory activities than steroidal glycoside 2, which possesses the Tig group at the C-11 position (in the previous study) [33] (Table S1). Hence, the different inhibitory activities of the identified compounds (steroid glycoside 3 (current research) and steroidal glycosides 1 and 2 (previous research) [33] may be because of the Ac group at the C-11 position, the dehydroxy alcohol and the hydroxy group at the C-8 position. Our findings indicate that the *M. tenacissima* leaves possess allelopathic activity, and its identified compounds, steroidal glycosides 1 and 2 (previous research) and steroid glycoside 3 (current research), may contribute to its allelopathy. Therefore, because of its allelopathic activity, *M. tenacissima* leaves may be used as mulch and a soil-additive resource to control weeds biologically as well as to protect the environment from the negative effects of commercial herbicides.

## 4. Materials and Methods

### 4.1. Plant Material

*Marsdenia tenacissima* leaves were collected from Khin-U Township, Shwe Bo district, Sagaing Division Region, Myanmar (22°49′4″ N and 95°48′12″ E) during July–August 2020 (Figure 1). The leaves were shade-dried and ground using an electric grinder. Before extraction, the leaf powder was kept in plastic bags in a refrigerator at 2 °C. Lettuce (*Lactuca sativa* L.), alfalfa (*Medicago sativa* L.), timothy (*Phleum pratense* L.), and barnyard grass (*Echinochloa crusgalli* (L.) Beauv.) were chosen as the test plants for growth bioassays.

### 4.2. Extraction and Growth Bioassay

Powdered leaf (500 g) was extracted with 3000 mL of 70% (*v/v*) aqueous methanol and then filtered through a single layer of filter paper (No. 2, 125 mm; Toyo Ltd., Tokyo, Japan) after being kept in the dark for 48 h. The extract residue was then re-extracted with methanol (3000 mL) for 24 h and filtered. The two filtrates were mixed and evaporated in a rotary evaporator to obtain crude extracts at 40 °C until dry. The crude extracts of *M. tenacissima* were dissolved in 300 mL of methanol to prepare six bioassay concentrations (1, 3, 10, 30, 100, and 300 mg dry weight (DW) equivalent extract/mL). To obtain those concentrations, an aliquot of the extract (0.45, 1.35, 4.5, 13.5, 45.11, and 135 µL, respectively) and control (100 µL methanol only) were added to sheets of filter paper (No. 2; Toyo Ltd.) in 28-mm Petri dishes. The Petri dishes were then placed in a fume hood to evaporate the methanol, and the filter papers were then moistened with 0.6 mL of a 0.05% aqueous solution of polyoxyethylene sorbitan monolaurate (Tween 20; Nacalai Tesque, Inc., Kyoto, Japan). Consequently, ten seeds of lettuce and alfalfa, and ten sprouted seeds of barnyard grass and timothy were placed on the filter papers and incubated in darkness at 25 °C for 36 h. Seedling length was measured and compared with the control seedlings to calculate the percentage of seedling growth after 48 h of incubation. A completely randomized design (CRD) with three replicates was used in the bioassay experiment, and the experiment for each test plant was repeated twice (10 seedlings/replicate, *n* = 60).

### 4.3. Purification of the Active Substance

The extraction method of *M. tenacissima* leaf powder (3500 g) and evaporation of the leaf extracts was carried out using the same method mentioned above. After evaporation of the aqueous methanol extracts, the aqueous residue was adjusted to pH 7.0 using 1 M phosphate buffer and then partitioned six times with an equal volume of ethyl acetate. The ethyl acetate fraction was then chromatographed on a column of silica gel, a column of Sephadex LH-20, reverse-phase C_18_ cartridges, and HPLC. The inhibited fractions in each isolation and purification step of silica gel, Sephadex LH-20, and reverse-phase C_18_ cartridges were similar to those used by Moh et al. (2022) [33]. In a reverse-phase C_18_ cartridge, the active fraction (F_6_) was obtained and evaporated using a rotary evaporator to acquire a crude residue. After that, one active substance in this fraction was purified by using reverse-phase HPLC (500 × 10 mm I.D., ODS AQ-325; YMC Ltd. Kyoto, Japan), eluted with 70% aqueous methanol at a flow rate of 1.5 mL/min, and detected at a wavelength of 220 nm and oven temperature of 40 °C. The peak fraction eluted during the retention time of 142–146 min included biological activity. This active peak fraction was purified again using reverse-phase HPLC (4.6 × 250 mm I.D., S-5 µm, Inertsil^®^ ODS-3; GL Science Inc., Tokyo, Japan), eluted with 70% aqueous methanol at a flow rate of 1.5 mL/min, Inhibitory activity of the active peak was discovered at a retention time of 52–57 min, resulting in one active compound. The molecular structure of the active compound was then characterized by HR-ESIMS, IR, ^1^H-NMR (400 MHz, CD_3_OD), and HMBC, TOCSY, NOESY, ^13^C-NMR spectrum (100 MHz, CD_3_OD), and optical rotations. HR-ESIMS spectra were obtained on an LCT Premier XE time-of-flight (TOF) mass spectrometer. A JASCO DIP-1000 polarimeter was used to quantify optical rotations. UV spectra were obtained using a JASCO V730-BIO spectrophotometer. A Bruker ALPHA instrument was used to record the IR spectra. All NMR spectral data were recorded on JEOL JNM-ECX400 and JNM-ECS400 spectrometers for ^1^H (400 MHz) and ^13^C (100 MHz).

### 4.4. Biological Activity of the Characterized Compound

The identified compound was dissolved in 2 mL of methanol. The assay concentrations of 0.01, 0.03, 0.1, 0.3, 1, 3, and 6 mM were then prepared and added (0.7, 2.1, 7.1, 21.4, 71.4, 214.3, and 428.57 µL, respectively, of compound solution) to sheets of filter paper (No. 2, 28 mm; Toyo) in 28 mm Petri dishes. The Petri dishes were dried in a fume hood and then moistened with 0.6 mL of Tween 20. Ten seeds of cress (*Lepidium sativum*) were placed on the filter papers in the Petri dishes and incubated under darkness at 25 °C for 48 h. The cress shoot and root lengths were measured to determine the percentage of seedling growth.

### 4.5. Statistical Analysis

Three replicates and two rounds of the bioassay experiments were carried out using a completely randomized block design (CRD). The results were presented as the mean ± standard error (SE). ANOVA of all the data was carried out using SPSS software, version 16.0 (SPSS Inc., Chicago, IL, USA), and significant differences between the control and sample treatments were determined using Tukey’s test at a significance level of 0.05. The *M. tenacissima* extracts and the related compound concentrations required for 50% growth inhibition (*I_50_* value) of the tested plants were calculated using Graph Pad Prism^®^ Ver. 6.0 (GraphPad Software, Inc., La Jolla, CA, USA).

## 5. Conclusions

The leaf extracts of *M. tenacissima* showed significant allelopathic potential against the seedling growth of lettuce, alfalfa, barnyard grass, and timothy. The identified allelopathic substance (active compound) was isolated and determined to be a novel compound, steroidal glycoside 3 (3-*O*-[*β*-cymaropyranosyl (1→4)-*β*-cymaropyranosyl]-8-dehydroxy-11*β*-*O*-acetyl-12*β*-*O*-tigloyl-17*β*-marsdenin). This compound significantly suppressed the shoot and root growth of cress. The growth inhibitory activities of this compound may be responsible for the allelopathic activity of *M. tenacissima* leaves. However, additional field study is required to validate the phytotoxic activity of *M. tenacissima* and to identify the mode of action of its active compound. Thus, our findings suggest that *M. tenacissima* leaves may have good weed control potential as mulch and a soil-additive resource, and its allelopathic compound may be considered a promising candidate for an ecofriendly herbicide to reduce the reliance on commercial herbicides in sustainable agriculture.

## Figures and Tables

**Table 1:** *I_50_* values (mg DW equivalent extract/mL) of the shoot and root growth of lettuce, alfalfa, barnyard grass, and timothy by the aqueous methanol extracts of *Marsdenia tenacissima*.

Test Plant		*I_50_* Value (mg DW Equivalent Extract/mL)	
		Shoot	Root
Dicots	Lettuce	3.8 ^d^	3.2 ^d^
	Alfalfa	3.5 ^d^	3.9 ^d^
Monocots	Barnyard grass	54.2 ^a^	12.6 ^db^
	Timothy	9.1 ^c^	0.7 ^e^

Different letters indicate significant difference according to Tukey’s HSD test (*p* < 0.05).

**Table 2:** NMR data of the active compound in CD_3_OD.

Position	δ_C_, (Type) ^a^	δ_H_ (*J* in Hz) ^b^	COSY/TOCSY	Selected HMBC	Selected NOESY
1a	39.0, CH_2_	1.22, m	2a, 2b		18
1b		1.56, m	2b		18
2a	30.7, CH_2_	1.28, m	1a, 1b, 3		
2b		1.77, m	1a, 1b, 3		
3	77.9, CH	3.61, m	2a, 2b, 4a, 4b		
4a	36.0, CH_2_	1.22, m	3, 5		
4b		1.66, m	3, 5		
5	45.7, CH	1.23, m	4a, 4b, 6a, 6b		
6a	30.2, CH_2_	1.32, m	5, 7a, 7b		
6b		1.38, m	5, 7a, 7b		
7a	29.1, CH_2_	1.16, m	6a, 6b, 8		
7b		2.13, m	6a, 6b, 8		
8	40.8, CH	1.77, m	7a, 7b, 9	14	
9	51.0, CH	1.45, m	8, 11		
10	38.7, C				
11	72.5, CH	5.82, dd (10.1, 10.1)	9, 12	1′	18, 19
12	78.7, CH	4.86, d (10.1)	11	1″	
13	55.6, C				
14	85.2, C				
15a	34.4, CH_2_	1.87, m	16a, 16b	14	
15b		2.11, m	16a, 16b	14	
16a	24.9, CH_2_	1.99, m	15a, 15b, 17		
16b		2.08, m	15a, 15b, 17		
17	59.0, CH	2.89, dd (4.8, 9.6)	16a, 16b		
18	12.6, CH_3_	0.94, s		1, 5, 9, 10	1a, 1b, 11
19	11.7, CH_3_	1.04, s		12, 13, 14, 17	11, 21
20	216.0, C				
21	32.0, CH_3_	2.12, s		17, 20	19
Acetyl					
1′	172.1, C				
2′	21.6, CH_3_	1.82, s		1′	
Tigloyl					
1″	169.1, C				
2″	129.1, C				
3″	140.6, CH	6.93, brq	4″	2″	
4″	14.6, CH_3_	1.85, brs	3″		
5″	12.1, CH_3_	1.85, s		1″, 2″	
Cym I					
1′″	98.6, CH	4.64, dd (1.6, 9.6)	2′″a, 2′″b	3	
2′″a	37.9, CH_2_	1.34, m	1′″, 3′″		
2′″b		2.23, m	1′″, 3′″		
3′″	80.5, CH	3.37, m	2′″, 4′″		
4′″	84.0, CH	3.17, m	3′″, 5′″		
5′″	72.5, CH	3.66, m	4′″, 6′″		
6′″	18.8, CH_3_	1.35, d (5.9)	5′″		
13′″	57.4, CH_3_	3.40, s		3′″	
Cym II					
7′″	102.2, CH	4.72, d (8.1)	8′″	4′″	
8′″	73.6, CH	3.31, m	7′″, 9′″		
9′″	84.0, CH	3.62, m	8′″, 10′″		
10′″	75.0, CH	3.17, m	9′″, 11′″		
11′″	71.3, CH	3.37, m	10′″, 12′″		
12′″	18.2, CH_3_	1.23, d (6.3)	11′″		
14′″	62.5, CH_3_	3.59, s		9′″	

^a^ Measured at 100 MHz. ^b^ Measured at 400 MHz. These are the abbreviation of NMR peak multipliacation and explained as d = doublet, dd = doublet of doublet, m = multiplet, s = singlet, brq = broad quarted, brs = broad singlet.

## Data Availability

Not applicable.

## Supplementary Materials

The following supplementary table can be downloaded at: https://www.mdpi.com/article/10.3390/plants12081663/s1, Table S1: *I_50_* value (mM) of the shoot and root growth of cress by steroidal glycosides 1, 2 and 3.
