# Supplementary material for: Allelopathic Potential of Marsdenia tenacissima (Roxb.) Moon against Four Test Plants and the Biological Activity of Its Allelopathic Novel Compound, 8-Dehydroxy-11β-O-Acetyl-12β-O-Tigloyl-17β-Marsdenin
Source: Plants (Basel). 2023 Apr 15;12(8):1663. doi: 10.3390/plants12081663 (PMC10142190; doi:10.3390/plants12081663)
Supplement: Supplementary file 1 [file plants-12-01663-s001.zip › plants-2292196-supplementary.pdf]

Table S1. I_{50} value (mM) of the shoot and root growth of cress by steroidal glycosides 1, 2, and 3.

**Test plant**		**I_{50} value (mM)**		
		**Steroidal glycoside 1**	**Steroidal glycoside 2**	**Steroidal glycoside 3**
Cress	Shoot	0.45 b	0.74 a	0.25 c
	Root	0.03 e	0.12 d	0.03 e

Different letters indicate significant difference according to Tukey's HSD test ($p < 0.05$).
